# Serological evidence of continued exposure to *Plasmodium falciparum* among residents in a near-elimination setting

**DOI:** 10.3389/fmicb.2025.1742072

**Published:** 2026-01-09

**Authors:** Muhammad Muzhaffar Daud, Aprillia Andika Rahayu, M. Ilyas, Derico Hitipeuw, Fadhila Fitriana, Jadidan Hada Syahada, Raisha Nuranindita, Fariha Akmalina Amirudin, Edo Yungki Saputra, Bo-Young Jeon, Ni Kadek Dita Cahyani, Nuruliarizki Shinta Pandupuspitasari, Jin-Hee Han, Fauzi Muh

**Affiliations:** 1 Department of Epidemiology and Tropical Diseases, Faculty of Public Health, Universitas Diponegoro, Semarang, Indonesia; 2 Public Health Genomics Lab, Faculty of Public Health, Universitas Diponegoro, Semarang, Indonesia; 3 Department of Tropical Medicine, School of Medicine, Kangwon National University, Chuncheon, Republic of Korea; 4 Department of Health Entomology, Faculty of Public Health, Universitas Diponegoro, Semarang, Indonesia; 5 Department of Biomedical Laboratory Science, College of Software and Digital Healthcare Convergence, Yonsei University, Wonju, Republic of Korea; 6 Department of Biology, Faculty of Science and Mathematics, Universitas Diponegoro, Semarang, Indonesia; 7 Department of Animal Science, Faculty of Animal and Agricultural Sciences, Universitas Diponegoro, Semarang, Indonesia

**Keywords:** antibody responses, exposed-individual, malaria, risk factors, serosurveillance

## Abstract

**Background:**

Malaria transmission has significantly declined in several endemic regions following extensive control efforts, bringing some areas close to elimination status. However, residual exposure to *Plasmodium falciparum* may persist even when clinical cases become rare. Serological markers provide valuable insights into historical and recent exposure by detecting long-lived antibodies that remain after infection. The use of blood-stage antigens such as PfEBA175 and PfRh5 allows for assessing population-level immunity and identifying potential hotspots of residual transmission.

**Methods:**

A community-based cross-sectional study was conducted from May to June 2025 across four endemic villages (Sedayu, Kemejing, Kembaran, and Wadas) in the Menoreh Hills. Blood samples from 120 malaria-exposed individuals and 24 malaria-negative controls were analyzed using protein microarray to assess antibody responses to PfEBA175 and PfRh5 antigens. Sociodemographic and behavioral risk factors were obtained through structured questionnaires.

**Results:**

Significant heterogeneity in antibody responses was observed across villages from two antigens. Seropositivity of PfEBA175 was higher than PfRh5. PfEBA175 seroprevalence ranged from 10 to 40%, with significantly higher responses in Kembaran (40%, *p* < 0.001) and Wadas (23%, *p* = 0.003) compared with controls, reflecting focalized transmission. PfRh5 demonstrated universal immunogenicity with 53–70% seroprevalence across all villages (*p* < 0.001), with Sedayu showing the highest responses (70%) linked to nocturnal livestock activities (*p* = 0.006). Village-specific risk factors including indoor biting exposure, previous malaria history, outdoor nighttime activities, educational level, and travel to endemic areas were significantly associated with antibody profiles, highlighting micro-epidemiological heterogeneity in malaria exposure.

**Conclusion:**

This study reveals substantial micro-epidemiological variation in naturally acquired immunity to blood-stage malaria antigens in a pre-elimination setting. PfRh5 demonstrated universally strong immunogenicity across transmission intensities, whereas PfEBA175 exhibited heterogeneous responses reflecting focal exposure shaped by behavioral and environmental factors. These complementary patterns may inform multi-antigen vaccine strategies, village-specific interventions in areas with residual malaria transmission, and sero-surveillance development.

## 1 Introduction

Despite notable progress achieved through decades of intensive control efforts, malaria remains one of the most significant and persistent global public health challenges. According to the World Health Organization’s 2024 World Malaria Report, an estimated 263 million malaria cases and 597,000 deaths occurred globally by 2023, with an increase of approximately 11 million cases compared to 2022, whereas the number of deaths remains almost constant (WHO, 2024). In addition to this substantial burden, recent evidence from studies in Uganda, Benin, Ghana, and Malawi further shows that malaria during pregnancy can adversely affect early child neurodevelopment, including cognitive, motor, behavioral, and language delays, some persisting up to 36 months (Nema et al., 2025). These long-term developmental impacts further reinforce the persistent challenges posed by malaria. Malaria continues to demonstrate remarkable resilience, with 2022 recording 234.8 million clinical cases of *Plasmodium falciparum* (Weiss et al., 2025). This persistent transmission, particularly in areas where conventional interventions have plateaued, underscores the urgent need for novel complementary tools to achieve the elimination goals.

A similar phenomenon has been observed in Indonesia, the world’s largest archipelagic nation with extraordinary ecological and epidemiological diversity, where malaria remains a significant public health concern despite substantial progress toward its elimination. Indonesia ranked second after India in terms of malaria cases, with 1.09 million cases reported in 2023 (WHO, 2024). Endemic malaria transmission occurs with all five *Plasmodium* species known to infect humans, along with 20 confirmed *Anopheles* vector species, creating a complex heterogeneous transmission landscape (Surjadjaja et al., 2016). Current surveillance data indicate that approximately 50% of malaria cases nationwide are caused by *P. falciparum*, 30% by *P. vivax*, 10% by *P. malariae*, and 10% by other species, which reveals that *P. falciparum* remains the predominant or co-dominant species in several endemic regions, particularly in eastern Indonesia and certain focal areas of Java and other islands (Aisyah et al., 2024).

Purworejo Regency, located in Java Island, exemplifies malaria elimination challenges, characterized by ongoing *P. falciparum* transmission that persists despite decades of control interventions. Over a three-year period, malaria cases in the Purworejo Regency exhibited a significant decline, decreasing from 582 cases in 2021 to 576 cases in 2022, and then markedly dropping to 49 cases in 2023, which constitutes a 91.6% reduction from the baseline. During this timeframe, *P. falciparum* remains the predominant species, consistently representing the majority of infections (>99% in 2021, 89% in 2022, and 100% of indigenous cases in 2023) (Office PH, 2022, 2024). Most malaria cases in the Purworejo Regency have been reported in five malaria-endemic sub-districts: Bener, Kaligesing, Loano, Bagelen, and Purworejo. Kaligesing Sub-district, situated in the Menoreh Hills, has the highest number of cases, resulting in the highest endemicity rate in Central Java Province (Office PH, 2024). The Menoreh Hills in Purworejo Regency present a unique malaria situation because dense forests, hilly terrain, and abundant water sources create an ideal habitat for *Anopheles* mosquitoes and increase residents’ exposure to mosquito bites (Rejeki et al., 2019).

Malaria vaccines represent a complementary tool within the broader elimination toolbox. While vector-control interventions such as insecticide-treated nets, indoor residual spraying, and environmental management remain the primary means of reducing transmission, vaccines add an additional layer of protection by directly enhancing individual immunity (Eme et al., 2025; Healer et al., 2017). Several malaria vaccine candidates targeting malaria parasites are under preclinical and clinical development and blood-stage vaccines has become the spotlight as it targets asexual erythrocytic stage (Miura, 2016; Ntege et al., 2017). In elimination settings, particularly in low-transmission areas, blood-stage vaccines may play an increasingly strategic role. When cases become rare, major challenges arise from sporadic, hard-to-detect infections and the risk of imported cases. By inhibiting parasite multiplication in the blood, blood-stage vaccines can potentially suppress parasitemia, preventing single infections from developing into new sources of transmission (De Graaf et al., 2021; Tham et al., 2012; Paul et al., 2016). In this comprehensive vaccine development landscape, serological studies play a vital and multifaceted role in evaluating vaccine candidate potential by measuring naturally acquired antibody responses in exposed populations, identifying immunological correlates of clinical protection, assessing population-level immunity to inform deployment strategies, and characterizing the breadth and durability of immune responses to specific antigens (Partey et al., 2018).

Among numerous *P. falciparum* blood-stage antigens investigated as potential vaccine candidates, PfEBA175 and PfRh5 have been identified as particularly promising based on converging evidence from functional biology, immunology, and preclinical vaccine studies (Sané et al., 2025a; Douglas et al., 2015; Wanaguru et al., 2013). Research has highlighted the immunogenicity of PfRh5, as it inhibits parasite growth *in vitro* and is associated with protection from clinical malaria, indicating their potential as vaccine targets (Chiu et al., 2014). The PfRh5 antigen is part of a protein complex that is essential for erythrocyte invasion, and IgG subclass responses, particularly IgG3, have been strongly linked to protective immunity in naturally exposed populations (Weaver et al., 2016). Conversely, PfEBA175, another merozoite antigen, is involved in the initial binding of parasites to erythrocytes. While specific data on PfEBA175 immunogenicity in the provided studies is not detailed, it is generally considered alongside other antigens in evaluating immune responses in malaria research (Partey et al., 2018). Taken together, these lines of evidence justify the selection of PfEBA175 and PfRh5, as both function as essential ligands for *P. falciparum* erythrocyte invasion: PfEBA175 through its conserved Duffy-binding-like (DBL)-mediated binding to glycophorin A (Wanaguru et al., 2013), and PfRh5 through its obligatory interaction with basigin via the PfRh5-CyRPA-RIPR complex (Reddy et al., 2015). Understanding population-level antibody responses to these key invasion ligands is therefore essential for clarifying their epidemiological relevance in malaria-endemic settings.

Serological surveillance is increasingly recognized as a sensitive tool for malaria elimination, as antibody responses can detect past exposure and uncover low-density or asymptomatic infections often missed by routine diagnostics (Mamnani et al., 2025). By estimating transmission intensity and identifying residual hotspots, serosurveillance enables more targeted interventions and helps prevent resurgence in low-transmission settings (Elliott et al., 2014; Byrne et al., 2022). Its operational value has been demonstrated through strategies such as the *P. vivax* serological testing-and-treatment (PvSeroTAT) model, which uses a defined antigen panel to identify recently exposed individuals likely to harbor dormant infections that require treatment. Although PvSeroTAT is *vivax*-specific, it illustrates how serology can detect hidden reservoirs that escape conventional diagnostics, a principle relevant to *P. falciparum* surveillance, where low-level transmission persists (Sacks et al., 2018). Therefore, this study aimed to investigate the seroprevalence of two specific antigens, PfEBA175 and PfRh5, in the continuously-exposed population within the Menoreh Hills region to uncover the hidden reservoir of infection that may contribute to continued transmission. The data collected on the prevalence of these antigens could provide valuable insights into future vaccine development, potentially targeting the most prevalent or immunogenic antigens in the local population.

## 2 Methods

### 2.1 Study site selection

**Figure 1:**
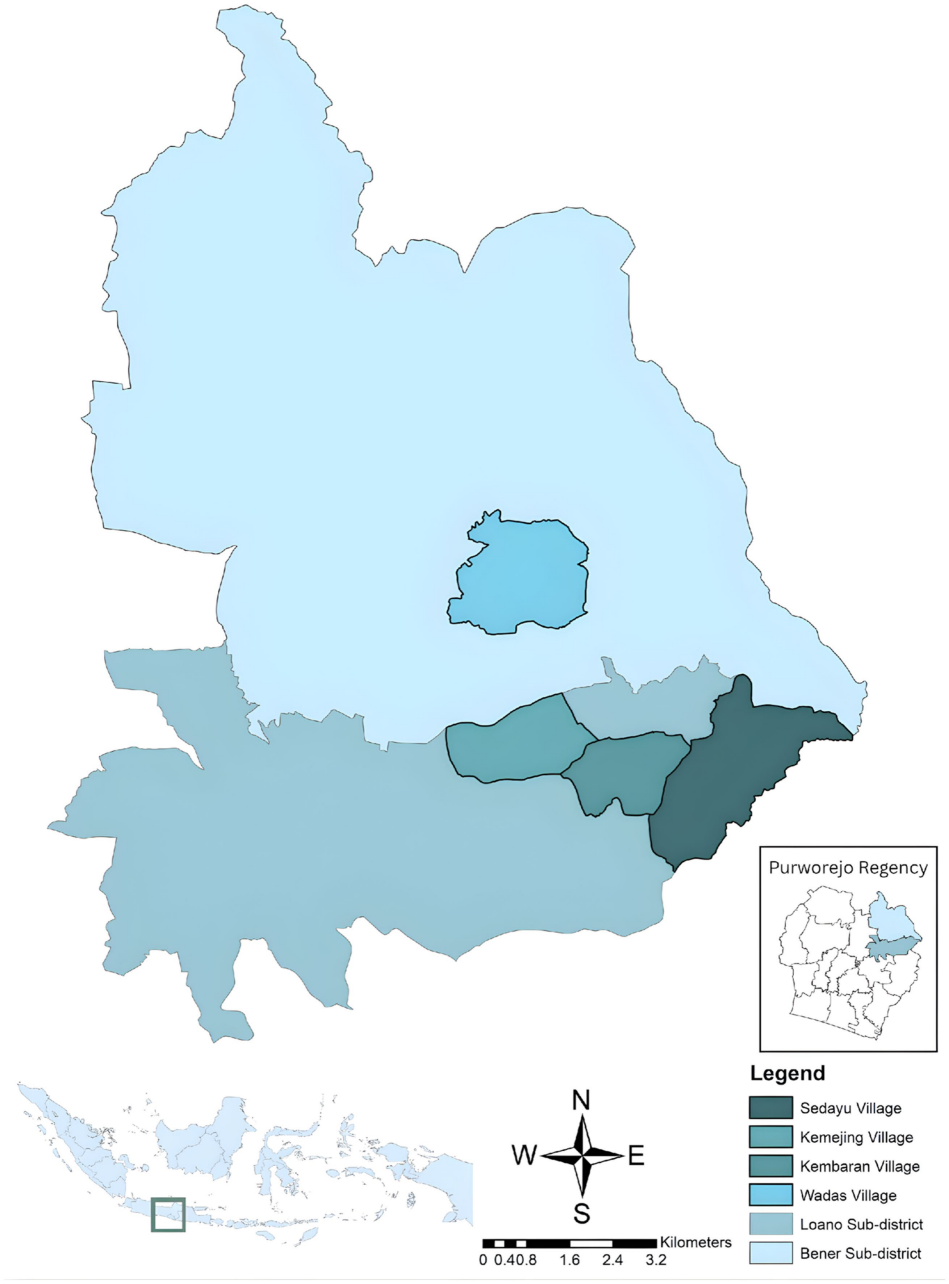
Map of the study sites located in the Menoreh Hills, Purworejo Regency, Central Java Province, comprising Sedayu village, Kemejing village, and Kembaran village in Loano sub-district, and Wadas village in Bener sub-district.

A community-based cross-sectional study was conducted from May to June 2025. Four endemic villages comprising Sedayu, Kemejing, Kembaran, and Wadas were purposely chosen based on topographic and ecological features that facilitated sustained malaria transmission (Figure 1). The Menoreh Hills region is a complex area consisting of interconnected forest ecosystems, an agricultural matrix, and diverse hydrological features. These environmental features provide favorable breeding sites for the different *Anopheles* species, which are the main carriers of *Plasmodium falciparum*.

### 2.2 Sample collections

The structured questionnaire was designed to obtain comprehensive information on various aspects related to malaria risk assessment. For the purpose of this study, exposed populations are defined as individuals living in malaria endemic area whose behaviors or living conditions increase their likelihood of contact with malaria vectors. Demographic details, including age, gender, occupation, and educational level, were gathered to provide a baseline understanding of the study population. The questionnaire of mosquito exposure behaviors, and malaria risk-related behaviors were assessed to reflect the potential of exposure to vector populations. Additionally, the survey included questions on respondents’ lifetime history of malaria, defined as whether they had ever been diagnosed with malaria, which may reflect prior exposure related to acquired immunity or susceptibility to future infections. Each study site was cross-sectionally surveyed.

A minimum of 99 samples was calculated using Cochran’s formula (Lubis et al., 2025), based on an estimated seroprevalence of 6.9% (Surendra et al., 2019) and an absolute precision of 5% with a 95% confidence interval (CI). Given that the study area currently represents a low-transmission, near-elimination setting, this prevalence was considered a conservative and appropriate estimate. To assess antigenicity, 30 participants were selected from each of the four study sites, resulting in a total sample size of 120. Participants were selected using a purposive sampling method following inclusion criteria that required individuals to have lived in the village or study site for more than 3 months, to be older than 1 year, having no comorbid of diabetes mellitus and HIV/AIDS, and also to be able to provide informed consent. Each individual voluntarily donated 2 mL of venous blood collected in an EDTA tube. Whole blood samples were taken if permitted by the participant or guardian. Sera was separated using a centrifugation method. The sera was stored in a dedicated −20 °C freezer until analysis. All clinical samples were collected in accordance with ethical guidelines and approved protocols from the Health Research Ethics Committee, Faculty of Public Health, Diponegoro University (No: 144/EA/KEPK-FKM/2025). Informed consent was obtained from all participants.

### 2.3 Recombinant protein expressions

The recombinant protein constructs were designed based on the *P. falciparum* 3D7 strain sequence (PfEBA175, PF3D7_0731500 (152–743 aa); PfRh5: PF3D7_0424100 (25-526aa)) resulting in an expected molecular weight of 81.2 and 64.4 kDa, respectively. This construct incorporated all relevant non-synonymous mutations necessary for antigenicity screening. The DNA fragments encoding those proteins were amplified from Pf3D7 with primer pairs PfEBA175_F: 5’-CTTGGAGCGGCCGCAAATAAGTACGTGCCCATCAACG-3′; PfEBA175_R: 5’-GTTCTCTGGCGCGCCGTCCTTCACTTCGGGGCA-3′; PfRh5_F: 5’-CCCATGAAGAATTGAGTCACATAATAGAATGAT-3′; PfRh5_R: 5’-CCATGTTTTGTCATTTCATTGTGTAAGTGGT-3′. Protein production was conducted using the HEK293 EBNA1-6E cell expression system (Miura, 2016). The codon-optimized genes were amplified with specific primers and subcloned into the pTT5-8x His expression vector using the In-Fusion HD Cloning Kit (Clontech, Mounta in View, CA, United States). Transfection of HEK293 EBNA1-6E cells was performed using linear polyethylenimine hydrochloride as a transfection reagent, and the secreted recombinant protein was harvested from the culture supernatant after 5 days. Purification was carried out using Ni-NTA agarose (QIAGEN) with an elution buffer containing 350 mM imidazole, 50 mM HEPES, 5% glycerol, and 150 mM NaCl. The purified protein (1 μg) was prepared with a reducing buffer and resolved using a 4–12% gradient Bis-Tris Mini Protein Gel (Invitrogen, Waltham, MA, United States). The gel was subsequently stained with Coomassie Brilliant Blue (Sigma-Aldrich) to confirm protein purity and integrity.

### 2.4 Antigenicity screening

To evaluate the antigenic variation of recombinant proteins, a protein microarray technique was employed. Antigens were systematically immobilized on aminopropyl-coated slides as previously described (Jun et al., 2024; Sané et al., 2025b). Briefly, the recombinant proteins were applied at an optimized concentration of 50–100 ng/μL per spot and incubated for 2 h at 37 °C to ensure proper antigen attachment. The slides were then blocked with a 5% bovine serum albumin (BSA) solution in phosphate-buffered saline with Tween 20 (PBS-T) for 1 h at 37 °C to minimize non-specific binding. Sera samples were collected from two distinct groups: 24 healthy individuals from South Korea with no history of malaria and 120 exposed-populations from four villages. Sera samples were diluted 1:25 in PBS-T and applied in duplicate to each antigen spot, followed by incubation for 1 h at 37 °C. The secondary antibody was detected using Alexa Fluor 546-conjugated goat anti-human IgG (10 ng/μL) in PBS-T, with a subsequent 1-h incubation at 37 °C. The microarrays were scanned using an InnoScan 300 (INNOPSYS, Carbonne, France). Positive antigenic responses were determined using a cut-off defined as the mean fluorescence intensity (MFI) of healthy controls plus two standard deviations. Normalized MFI values were calculated as MFI divided by this cut-off.

### 2.5 Data analysis

The sociodemographics and behavioral characteristics were analyzed descriptively and analytically. Age was categorized into productive (15–64 years) and non-productive (<15 or >64 years) groups, in accordance with World Health Organization (WHO) standards. Educational level was classified as low (below senior high school) or high (completion of senior high school or higher). Occupation was grouped based on the primary work environment, with outdoor occupations defined as jobs predominantly performed outside (e.g., farmers, laborers, and construction workers), and indoor occupations defined as roles primarily conducted indoors (e.g., housewives, entrepreneurs, village officials, private-sector employees, village heads, pensioners, and merchants). History of malaria infection and travel history were assessed by asking participants whether they had ever been diagnosed with malaria within the last 5 years and whether they had ever traveled to malaria-endemic areas within the last 30 days. The protein microarray data were comprehensively analyzed and visualized using GraphPad Prism v10 (GraphPad Software, San Diego, CA, United States). Protein array data were first assessed for normality on MFI values per village using the Shapiro–Wilk test. Data were deemed normally distributed when the *p*-value exceeded 0.05 (95% confidence interval). Subsequent comparisons and associations between variables were conducted using an independent t-test for normally distributed data, or the Mann–Whitney U test for data that deviated from normality. Statistical significance was set at *p* < 0.05 (95% confidence interval). For serological analysis, participants were classified into exposed populations (individuals residing in malaria-endemic villages) and healthy controls (individuals from non-endemic areas with no history of malaria). Serological outcomes were operationalized as seropositivity, defined as the proportion of samples exceeding the antibody response threshold, and normalized MFI, representing the quantitative strength of antigen-specific antibody binding.

## 3 Results

### 3.1 Recombinant protein expressions and purifications

**Figure 2:**
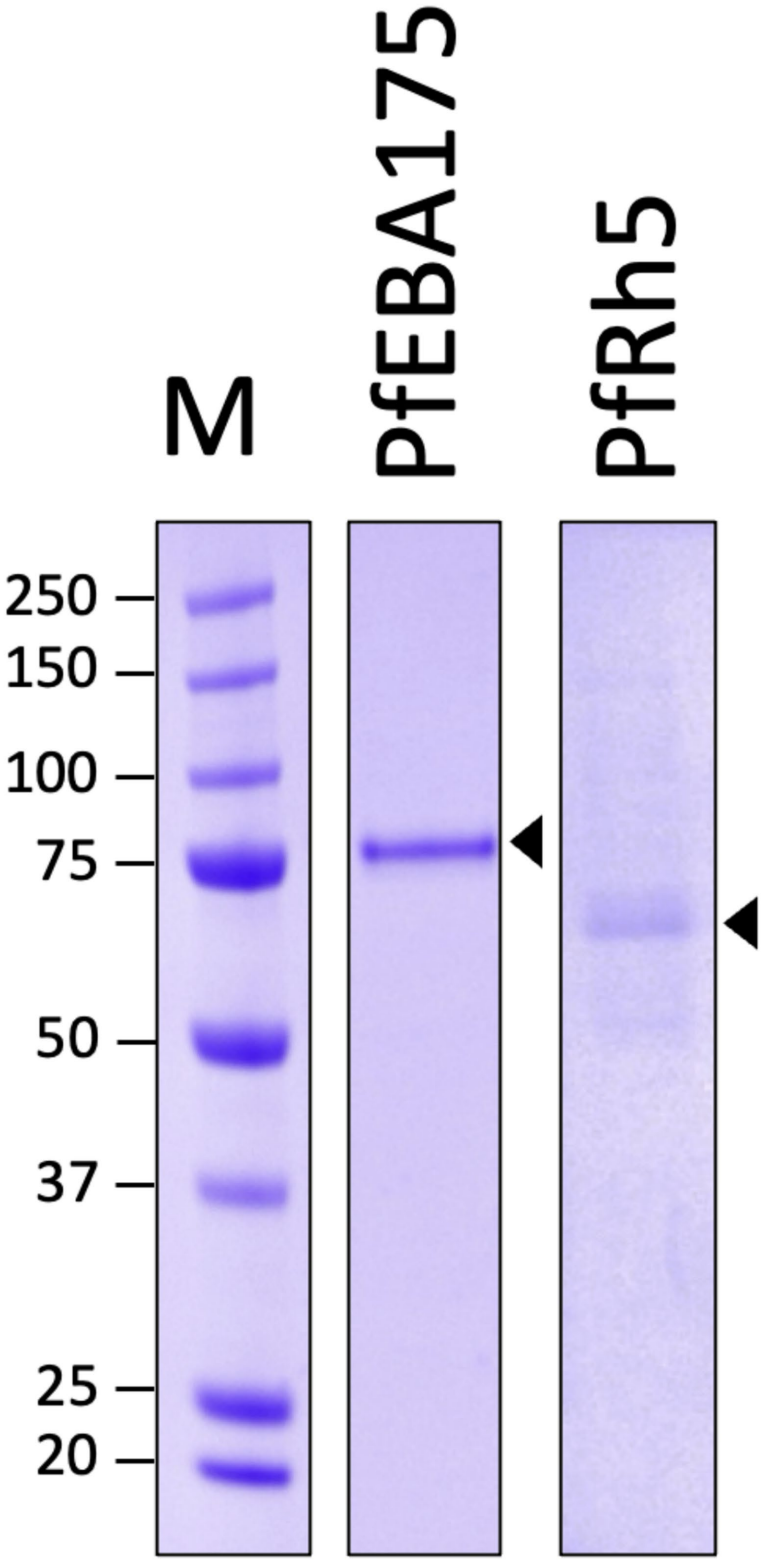
Purified *Plasmodium falciparum* antigens EBA175 and Rh5 used for antigenicity screening. PfEBA175, 81.2 kDa; PfRh5, 64.4 kDa; M, marker.

The SDS-PAGE analysis of the purified recombinant proteins is presented in Figure 2. The gel image shows distinct bands corresponding to the two *Plasmodium falciparum* proteins: PfEBA175 and PfRh5. The molecular weight marker (M) is shown in the leftmost lane, providing a reference for size estimation in kDa. Recombinant protein of PfEBA175 appears as a prominent band at approximately 81.2 kDa, consistent with its expected molecular weight. PfRh5 recombinant protein is visible as a distinct band at around 64.4 kDa. The presence of these well-defined bands at their respective molecular weights indicates successful expression and purification of the target proteins. The absence of significant additional bands suggests high purity of the isolated recombinant proteins (Figure 2).

### 3.2 Antigenicity screening

**Figure 3:**
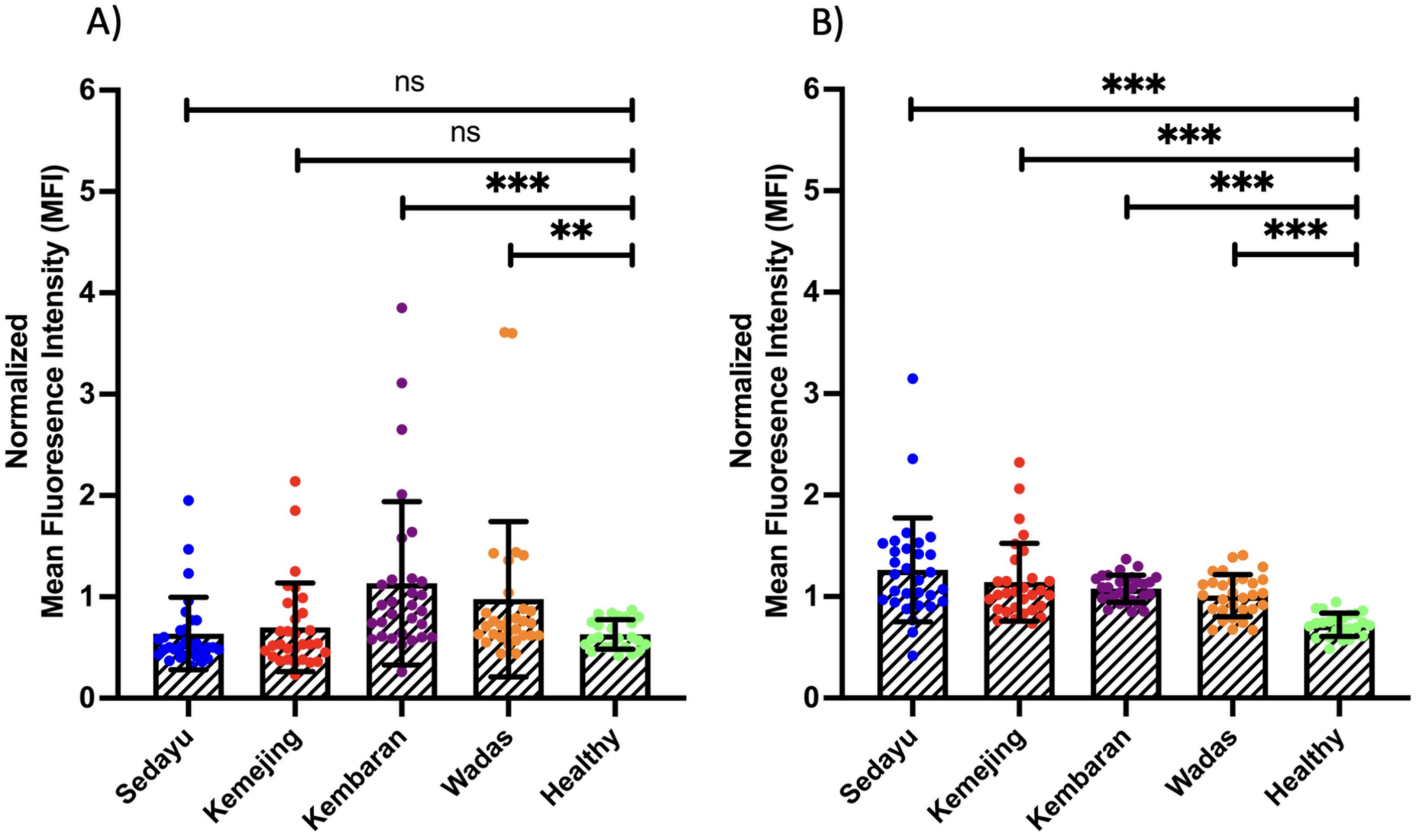
Antigenicity differences of two recombinant proteins, **(A)** *Pf*EBA175 and **(B)** *Pf*Rh5. Asterisks indicate statistical significance (** *p* < 0.01; *** *p* < 0.001); ns, not significant.

To assess naturally acquired antibody responses to two recombinant proteins, sera from exposed-individuals across four endemic villages in the Menoreh Hills (Sedayu, Kemejing, Kembaran, and Wadas) were shown as normalized MFI for each protein (Figure 3). For PfEBA175 (Figure 3A), significant differences were observed between the endemic villages and healthy controls, with Kembaran (12/30; 40%) showing the highest antibody responses. However, Wadas (7/30; 23%), Sedayu (3/30; 10%) and Kemejing (5/30; 17%) demonstrated lower responses, but still higher than the healthy controls (Table 1). Statistically significant differences were observed (*p* < 0.01 or *p* < 0.001), except for Sedayu and Kemejing, which were not significantly different from the healthy controls. This pattern reflects a gradient of naturally acquired exposure or immune stimulation to PfEBA175 across the endemic villages. The significant differences in PfEBA175 seropositivity rates observed for Kembaran and Wadas compared to healthy controls may support the specificity of these antibody responses to malaria exposure. In contrast, the lack of a significant difference for Sedayu may indicate lower transmission intensity or reduced exposure to PfEBA175 antigen in this village.

**Table 1:** Seropositivity rate of PfEBA175 recombinant proteins among exposed-populations and healthy controls across four study sites.

Villages	No. of exposed populations (*n*)			Normalized MFI	95% CI	No. of healthy samples (*n*)			Normalized MFI	95% CI	*p*-value
	Pos (*n*, %)^a^	Neg (*n*, %)	Total (*n*, %)			Pos (*n*, %)	Neg (*n*, %)^b^	Total (*n*, %)			
Sedayu	3 (10)	27 (90)	30 (100)	0.64	0.50–0.77	1 (4)	23 (96)	24 (100)	0.65	0.58–0.72	0.060
Kemejing	5 (17)	25 (83)	30 (100)	0.70	0.54–0.86	1 (4)	23 (96)	24 (100)	0.65	0.58–0.72	0.329
Kembaran	12 (40)	18 (60)	30 (100)	1.13	0.83–1.43	1 (4)	23 (96)	24 (100)	0.61	0.52–0.69	<0.001
Wadas	7 (23)	23 (77)	30 (100)	0.98	0.69–1.26	1 (4)	23 (96)	24 (100)	0.61	0.52–0.69	0.003

MFI, Mean fluorescence intensity; CI, Confidence intervals. ^a^Seropositive rate: % of positive in exposed-population samples. ^b^Seronegative rate: % of negative in healthy samples.

In contrast, PfRh5 (Figure 3B) showed uniformly strong statistical differences (*p* < 0.001) between all endemic villages and the healthy controls. Seropositivity rates ranged from 53 to 70% across the four endemic villages, with Sedayu and Kembaran showing the highest proportions (21/30; 70%), followed by Kemejing (18/30; 60%) and Wadas (16/30; 53%). These findings indicate substantial naturally acquired exposure to PfRh5 antigen in all endemic communities. The consistently higher seropositivity rates in the exposed populations compared with those in the healthy controls further support a clear distinction in PfRh5-specific immune responses associated with malaria exposure (Table 2).

**Table 2:** Seropositivity rate of PfRh5 recombinant proteins among exposed-populations and healthy controls across four study sites.

Villages	No. of exposed populations (*n*)			Normalized MFI	95% CI	No. of healthy samples (*n*)			Normalized MFI	95% CI	*p*-value
	Pos (*n*, %)^a^	Neg (*n*, %)	Total (*n*, %)			Pos (*n*, %)	Neg (*n*, %)^b^	Total (*n*, %)			
Sedayu	21 (70)	9 (30)	30 (100)	1.26	1.07–1.45	1 (4)	23 (96)	24 (100)	0.75	0.70–0.80	< 0.001
Kemejing	18 (60)	12 (40)	30 (100)	1.14	0.99–1.28	1 (4)	23 (96)	24 (100)	0.75	0.70–0.80	< 0.001
Kembaran	21 (70)	9 (30)	30 (100)	1.08	1.02–1.12	0 (0)	24 (100)	24 (100)	0.69	0.62–0.75	< 0.001
Wadas	16 (53)	14 (47)	30 (100)	1.01	0.93–1.08	0 (0)	24 (100)	24 (100)	0.69	0.62–0.75	< 0.001

MFI, Mean fluorescence intensity; CI, Confidence intervals. ^a^Seropositive rate: % of positive in exposed-population samples. ^b^Seronegative rate: % of negative in healthy samples.

### 3.3 Sociodemographic and behavioral characteristics

The sociodemographic characteristics presented in Table 3 provide an overview of potential risk factors for malaria exposure across four endemic villages in Purworejo. The majority of participants across all sites are of productive age (15–64 years old) (76.67–86.67%), which may be associated with a higher likelihood of outdoor activities and increased exposure to mosquito vectors. Gender distribution is relatively balanced, with slight variations between sites. Educational levels differ notably, with Sedayu having a higher proportion of highly educated individuals (43.33%) compared to the other villages (10–20%). This disparity in education could influence awareness and adoption of preventive measures against malaria. Occupation types vary among villages, with Kembaran having the highest percentage of indoor workers (66.67%), whereas the other villages show a more balanced distribution between indoor and outdoor occupations. The higher proportion of outdoor workers in Sedayu, Kemejing, and Wadas (56.67% at each site) may lead to increased exposure to malaria vectors. These sociodemographic factors may influence malaria exposure and subsequently affect the antibody profiles of individuals in these endemic areas, with variations in risk shaped by age, education, and occupational exposure to mosquito-prone environments.

**Table 3:** Statistical analysis of PfEBA175 antibody levels with sociodemographic characteristics among exposed-populations across four study sites.

Sites	Sedayu			Kemejing			Kembaran			Wadas		
	Total, *n* (%)	nMFI	*p*-value^a^	Total, *n* (%)	nMFI	*p*-value^a^	Total, *n* (%)	nMFI	*p*-value^a^	Total, *n* (%)	nMFI	*p*-value^a^
Age												
Productive	23(76.67)	0.61	1.000	24(80.00)	0.74	0.604	23(76.67)	1.23	0.292	26(86.67)	0.90	0.272
Nonproductive	7(23.33)	0.72		6(20.00)	0.53		7(23.33)	0.81		4(13.33)	1.47	
Gender												
Male	15(50.00)	0.63	0.065	15(50.00)	0.60	0.836	13(43.33)	1.32	0.477	17(56.67)	1.23	0.010
Female	15(50.00)	0.64		15(50.00)	0.80		17(56.67)	0.99		13(43.33)	0.65	
Educational Level												
Low	17(56.67)	0.71	0.802	27(90.00)	0.74	0.012	27(90.00)	1.18	0.269	24(80.00)	1.09	0.001
High	13(43.33)	0.54		3(10.00)	0.36		3(10.00)	0.74		6(20.00)	0.54	
Occupation												
Outdoor	17(56.67)	0.74	0.197	17(56.67)	0.78	0.054	10(33.33)	1.42	0.113	17(56.67)	1.24	0.001
Indoor	13(43.33)	0.55		13(43.33)	0.60		20(66.67)	0.99		13(43.33)	0.63	

^a^Nonparametric test, calculated with Mann–Whitney test. nMFI, mean of normalized MFI.

The behavioral characteristics of participants across four endemic villages in Purworejo (Sedayu, Kemejing, Kembaran, and Wadas) reveal varying levels of potential malaria exposure (Table 4). Mosquito bites during outdoor activities at night were reported by 33.33 to 56.67% of participants, with Sedayu having the highest percentage. Indoor mosquito bites were more prevalent, ranging from 46.67 to 83.33%, with Kembaran showing the highest incidence. Nighttime livestock-related activities, which could increase exposure risk, were relatively low across all villages. The use of protective measures varied; mosquito repellent usage was low (6.67 to 23.33%), while mosquito net usage ranged from 10.00 to 60.00%, with Kembaran showing the highest adoption. Travel history to endemic areas varied from 10.00 to 40.00%, potentially contributing to malaria exposure. Notably, the history of malaria infection differed significantly among villages, ranging from 30.00% in Wadas to 73.33% in Kembaran. These behavioral patterns and historical data suggest varying levels of malaria exposure risk across the villages, which may correlate with differences in antibody profiles. The higher rates of reported mosquito bites, lower use of protective measures, and higher history of malaria infection in some villages may potentially be associated with elevated antibody levels against malaria parasites.

**Table 4:** Statistical analysis of PfEBA175 antibody levels with behavioral characteristics among exposed-populations across four study sites.

Sites	Sedayu			Kemejing			Kembaran			Wadas		
	Total, *n* (%)	nMFI	*p*-value^a^	Total, *n* (%)	nMFI	*p*-value^a^	Total, *n* (%)	nMFI	*p*-value^a^	Total, *n* (%)	nMFI	*p*-value^a^
Frequently bitten by mosquitoes during outdoor activities at night												
Yes	17(56.67)	0.52	0.002	10(33.33)	0.86	0.015	15(50.00)	0.87	0.263	10(33.33)	0.89	0.210
No	13(43.33)	0.79		20(66.67)	0.62		15(50.00)	1.39		20(66.67)	1.15	
Frequently bitten by mosquitoes at home												
Yes	21(70.00)	0.56	0.113	23(76.67)	0.64	0.105	25(83.33)	1.20	0.278	14(46.67)	1.23	0.134
No	9(30.00)	0.82		7(23.33)	0.90		5(16.67)	0.79		16(53.33)	0.76	
Feeding livestock at night												
Yes	4(13.30)	0.87	0.903	3(10.00)	0.92	0.067	3(10.00)	1.06	0.809	6(20.00)	1.34	0.132
No	26(86.70)	0.60		27(90.00)	0.67		27(90.00)	1.14		24(80.00)	0.89	
Checking livestock at night												
Yes	3(10.00)	1.03	0.316	3(10.00)	0.92	0.067	2(6.67)	1.35	0.212	2(6.67)	1.10	0.228
No	27(90.00)	0.59		27(90.00)	0.67		28(93.33)	1.11		28(93.33)	0.97	
Use of repellent												
No	28(93.33)	0.65	0.677	28(93.33)	0.72	0.088	23(76.67)	1.21	0.607	25(83.33)	0.89	0.358
Yes	2(6.67)	0.49		2(6.67)	0.37		7(23.33)	0.86		5(16.67)	1.42	
Use of mosquito nets												
No	20(66.67)	0.58	0.644	21(70.00)	0.68	0.821	12(40.00)	1.14	0.626	27(90.00)	0.89	0.201
Yes	10(33.33)	0.76		9(30.00)	0.75		18(60.00)	1.13		3(10.00)	1.72	
Travel history to malaria endemic areas												
Yes	6(20.00)	0.56	0.132	5(16.67)	0.93	0.015	12(40.00)	0.88	0.597	3(10.00)	1.59	0.782
No	24(80.00)	0.66		25(83.33)	0.65		18(60.00)	1.30		27(90.00)	0.91	
History of malaria infection												
Yes	11(36.67)	0.58	0.491	16(53.33)	0.65	0.835	22(73.33)	1.12	0.467	9(30.00)	1.53	0.020
No	19(63.33)	0.67		14(46.67)	0.75		8(26.67)	1.18		21(70.00)	0.74	

^a^Nonparametric test, calculated with Mann–Whitney test. nMFI, mean of normalized MFI.

### 3.4 Statistical analysis of sociodemographic characteristics and antibody responses

Analysis of sociodemographic characteristics revealed that PfEBA175 antibody levels were generally not associated with age group, gender, educational level, or occupation across the four study villages (Table 3). Age showed no significant differences in Sedayu, Kemejing, Kembaran, or Wadas (all *p* > 0.05). Gender likewise demonstrated non-significant results in Sedayu, Kemejing, and Kembaran (all *p* > 0.05); however, males in Wadas exhibited significantly higher antibody levels than females (*p* = 0.010). For educational level, no significant associations were observed in Sedayu or Kembaran (both *p* > 0.05), whereas significant associations were detected in Kemejing (*p* = 0.012), where individuals with higher education exhibited lower antibody levels, and in Wadas (*p* = 0.001), where participants with lower educational attainment showed higher PfEBA175 responses. Occupational category also showed no significant associations in Sedayu, Kemejing, or Kembaran (all *p* > 0.05), while Wadas demonstrated a significant association (*p* = 0.001), with higher antibody levels among individuals engaged in outdoor occupations.

For PfRh5, antibody levels showed no significant associations with any sociodemographic factor in any of the study villages (Table 5). Age group, gender, educational level, and occupation all yielded non-significant results across Sedayu, Kemejing, Kembaran, and Wadas (all *p* > 0.05). These findings indicate that sociodemographic characteristics are not major determinants of PfRh5 antibody variability. In contrast, the limited site-specific associations observed for PfEBA175 suggest that localized exposure intensity, rather than demographic structure, may exert a more substantial influence on the magnitude of antibody responses.

**Table 5:** Statistical analysis of PfRh5 antibody levels with sociodemographic characteristics among exposed-populations across four study sites.

Sites	Sedayu			Kemejing			Kembaran			Wadas		
	Total, *n* (%)	nMFI	*p*-value^a^	Total, *n* (%)	nMFI	*p*-value^a^	Total, *n* (%)	nMFI	*p*-value^b^	Total, *n* (%)	nMFI	*p*-value^b^
Age												
Productive	23(76.67)	1.30	0.641	24(80.00)	1.17	0.736	23(76.67)	1.07	0.595	26(86.67)	1.00	0.415
Nonproductive	7(23.33)	1.14		6(20.00)	1.01		7(23.33)	1.10		4(13.33)	1.09	
Gender												
Male	15(50.00)	1.34	0.852	15(50.00)	1.06	0.245	13(43.33)	1.06	0.458	17(56.67)	1.04	0.398
Female	15(50.00)	1.19		15(50.00)	1.22		17(56.67)	1.09		13(43.33)	0.97	
Educational level												
Low	17(56.67)	1.14	0.917	27(90.00)	1.07	0.062	27(90.00)	1.08	0.557	24(80.00)	1.04	0.087
High	13(43.33)	1.42		3(10.00)	1.80		3(10.00)	1.03		6(20.00)	0.88	
Occupation												
Outdoor	17(56.67)	1.13	0.280	17(56.67)	1.09	0.516	10(33.33)	1.07	0.792	17(56.67)	1.04	0.418
Indoor	13(43.33)	1.37		13(43.33)	1.21		20(66.67)	1.08		13(43.33)	0.97	

^a^Nonparametric test, calculated with Mann–Whitney test. ^b^Parametric test, calculated with Independent *t*-test. nMFI, mean of normalized MFI.

### 3.5 Statistical analysis of behavioral characteristics and antibody responses

Behavioral characteristics showed mixed patterns of PfEBA175 antibody levels across the study villages (Table 4). Frequent mosquito bites during outdoor activities were significantly associated with higher PfEBA175 levels in Sedayu (*p* = 0.002) and Kemejing (*p* = 0.015); however, no significant associations were found in Kembaran or Wadas (*p* > 0.05). Most other behavioral variables, including frequent bites at home, feeding livestock at night, checking livestock at night, use of repellents, use of mosquito nets, travel history, and prior malaria infection, were not significantly associated with PfEBA175 levels in most villages (all *p* > 0.05). The exceptions were travel history in Kemejing (*p* = 0.015) and a history of malaria infection in Wadas (*p* = 0.020). Overall, these findings indicate that the behavioral determinants of PfEBA175 reactivity are limited and site-specific.

For PfRh5, the behavioral characteristics had minimal influence on antibody variability (Table 6). Most behavioral variables showed non-significant associations across the study sites (all *p* > 0.05), with two isolated exceptions: frequent mosquito bites at home in Wadas (*p* = 0.039) and checking livestock at night in Sedayu (*p* = 0.006). Despite these exceptions, the overall pattern suggests that behavioral characteristics are not major drivers of PfRh5 antibody variability. Taken together, the lack of consistent associations across both proteins suggests that inter-village differences in antibody responses are more likely driven by local malaria transmission intensity, heterogeneous exposure patterns, or underlying microepidemiological conditions rather than by individual behavioral factors.

**Table 6:** Statistical analysis of PfRh5 antibody levels with behavioral characteristics among exposed-populations across four study sites.

Sites	Sedayu			Kemejing			Kembaran			Wadas		
	Total, *n* (%)	nMFI	*p*-value^a^	Total, *n* (%)	nMFI	*p*-value^a^	Total, *n* (%)	nMFI	*p*-value^b^	Total, *n* (%)	nMFI	*p*-value^b^
Frequently bitten by mosquitoes during outdoor activities at night												
Yes	17(56.67)	1.01	0.187	10(33.33)	1.01	0.129	15(50.00)	1.04	0.121	10(33.33)	0.98	0.627
No	13(43.33)	1.20		20(66.67)	1.20		15(50.00)	1.12		20(66.67)	1.02	
Frequently bitten by mosquitoes at home												
Yes	21(70.00)	1.15	0.288	23(76.67)	1.15	0.606	25(83.33)	1.08	0.651	14(46.67)	1.09	0.039
No	9(30.00)	1.12		7(23.33)	1.12		5(16.67)	1.05		16(53.33)	0.94	
Feeding livestock at night												
Yes	4(13.30)	2.00	0.067	3(10.00)	1.06	0.556	3(10.00)	1.01	0.367	6(20.00)	1.05	0.649
No	26(86.70)	1.15		27(90.00)	1.15		27(90.00)	1.08		24(80.00)	1.00	
Checking livestock at night												
Yes	3(10.00)	2.37	0.006	3(10.00)	1.06	0.556	2(6.67)	0.95	0.167	2(6.67)	0.98	0.839
No	27(90.00)	1.14		27(90.00)	1.15		28(93.33)	1.09		28(93.33)	1.01	
Use of repellent												
No	28(93.33)	1.28	0.280	28(93.33)	1.15	0.868	23(76.67)	1.07	0.780	25(83.33)	0.98	0.126
Yes	2(6.67)	0.97		2(6.67)	1.04		7(23.33)	1.09		5(16.67)	1.14	
Use of mosquito nets												
No	20(66.67)	1.19	0.312	21(70.00)	1.19	0.330	12(40.00)	1.02	0.102	27(90.00)	1.00	0.298
Yes	10(33.33)	1.41		9(30.00)	1.01		18(60.00)	1.11		3(10.00)	1.13	
Travel history to malaria endemic areas												
Yes	6(20.00)	1.41	0.437	5(16.67)	0.98	0.148	12(40.00)	1.08	0.996	3(10.00)	1.13	0.298
No	24(80.00)	1.22		25(83.33)	1.17		18(60.00)	1.08		27(90.00)	1.00	
History of malaria infection												
Yes	11(36.67)	1.53	0.061	16(53.33)	1.14	0.950	22(73.33)	1.11	0.059	9(30.00)	1.05	0.496
No	19(63.33)	1.11		14(46.67)	1.14		8(26.67)	1.00		21(70.00)	0.99	

^a^Nonparametric test, calculated with Mann–Whitney test. ^b^Parametric test, calculated with Independent *t*-test. nMFI, mean of normalized MFI.

## 4 Discussion

This study provides comprehensive serological evidence of continued *Plasmodium falciparum* exposure in a near-elimination setting by simultaneously assessing antibody responses to PfEBA175 and PfRh5. Despite their shared biological function, these antigens revealed both concordant and contrasting patterns across the study population, PfEBA175 exhibited focalized antigenicity, with significant differences from non-endemic controls only in Kembaran (40%, *p* < 0.001) and Wadas (23%, *p* = 0.003), while Sedayu (10%, *p* = 0.060) and Kemejing (17%, *p* = 0.329) showed no significant difference. Conversely, PfRh5 responses were broadly elevated across all villages, with seropositivity of 70% in Sedayu and Kembaran, 60% in Kemejing, and 53% in Wadas; all showed highly significant differences versus controls (*p* < 0.001). These findings align with studies from Burkina Faso, Somalia, Peru, Malawi, and Zimbabwe demonstrating that micro-epidemiological heterogeneity in antibody profiles is driven by differences in entomological, environmental, and behavioral factors (Mabaso et al., 2006; Kabaghe et al., 2018; Bautista et al., 2007; Bousema et al., 2010; Guelbéogo et al., 2018).

Based on these patterns, malaria risk varied substantially across villages. Kembaran exhibited the highest transmission risk (PfEBA175: 40%, PfRh5: 70%). Wadas showed high-moderate risk (PfEBA175: 23%, PfRh5: 53%). Sedayu presented a paradoxical profile with minimal prevalence of PfEBA175 but elevated prevalence of PfRh5 (10%; 70%, respectively). Kemejing had the lowest transmission risk (PfEBA175: 17%, PfRh5: 60%). Risk factor analysis revealed PfEBA175 is responsive to current behavioral and demographic factors (Tables 3, 4), while PfRh5 shows broad elevation regardless of individual characteristics as most statistical analyses yielded non-significant results (Tables 5, 6). PfRh5 showed only two significant associations: checking livestock at night in Sedayu (*p* = 0.006) and indoor mosquito biting in Wadas (*p* = 0.039). Male gender was a significant risk factor for PfEBA175 in Wadas (*p* = 0.010), with males showing higher antibody levels (nMFI 1.23) than females (nMFI 0.65), that may suggest gender-specific activities and occupational exposures contribute to differential exposure. Educational attainment showed strong inverse associations with PfEBA175. In Wadas, individuals with lower education exhibited substantially higher reactivity (nMFI 1.09 vs. 0.54, *p* = 0.001), and in Kemejing, lower education associated with elevated levels (nMFI 1.72 vs. 0.36, *p* = 0.012). This pattern appeared consistently across all villages. Greater education likely translates into improved prevention practices, better housing, and reduced exposure (Alao et al., 2025; Mohan et al., 2021; St Laurent et al., 2016). Meanwhile, educational level showed no consistent association with PfRh5.

Outdoor work was a significant determinant of PfEBA175 in Wadas (*p* = 0.001), with outdoor workers showing elevated levels (nMFI 1.24 vs. indoor 0.63). This pattern was consistent across villages. Outdoor occupations involve activities during peak mosquito activity hours with less access to protective environments (Makenga et al., 2025). No significant associations were found between occupation and PfRh5 (*p* > 0.280). Previous malaria infection was significantly associated with PfEBA175 in Wadas (*p* = 0.020), where individuals with past infections showed higher levels (nMFI 1.53 vs. 0.74), likely reflecting greater historical exposure and immune memory boosting. In Kembaran, where 73.33% had malaria history, no significant association was found (*p* = 0.467), likely because near-universal prevalence reduced variability. For PfRh5, malaria history showed a near-significant trend in Kembaran (*p* = 0.059).

Frequent indoor mosquito biting was significantly associated with PfRh5 in Wadas (*p* = 0.039), where individuals reporting frequent biting showed higher levels (MFI = 1.09 vs. 0.94). This was one of only two factors showing significant PfRh5 association, highlighting indoor vector contact’s importance. For PfEBA175, indoor biting showed consistent trends but no significance: Kembaran (83.33% reported, nMFI 1.20 vs. 0.79, *p* = 0.278) and Wadas (nMFI 1.23 vs. 0.76, *p* = 0.134). Checking or tending livestock at night emerged as the strongest risk factor, showing highly significant associations with PfRh5 in Sedayu (*p* = 0.006). Though only 10% engaged in this activity, they showed dramatically elevated responses (nMFI 2.37 vs. 1.14). This activity exposes individuals to peak Anopheles feeding hours near animal shelters with high vector densities, while unprotected by nets or repellents, creating a high-risk window distinct from household exposure (Rozi et al., 2022; Mwalugelo et al., 2024; Mohan et al., 2021).

Travel to endemic regions was significantly associated with PfEBA175 in Kemejing (*p* = 0.015), where travelers showed elevated responses (nMFI 0.93 vs. 0.65), underscoring human mobility’s role in sustaining antibody responses in near-elimination zones (Muller et al., 2025). Travel history to malaria endemic areas showed no significant association with PfRh5 (*p* > 0.05 for all villages). This may be in line with their time of travel at day time (7 a.m. to 4 p.m.). Outdoor mosquito biting showed significant association with PfEBA175 in Kemejing (*p* = 0.015), where participants demonstrated elevated levels (nMFI 0.86 vs. 0.62), consistent with peak Anopheles biting exposure (Sarrassat et al., 2025). Interestingly, Sedayu showed a reversed association (*p* = 0.002), where participants had lower PfEBA175 levels (nMFI 0.52 vs. 0.79), possibly because outdoor workers avoid homes during indoor peak biting. No significant associations with PfRh5 were observed (*p* > 0.05 for all villages).

Beyond significant associations, several descriptive patterns warrant consideration. Protective measure use was nearly universally low: 76–93% did not use repellents, 40–90% did not use mosquito nets (Table 4). These conditions may facilitate increased indoor mosquito exposure, as documented in previous studies (Esayas et al., 2024; Wilson et al., 2014). Poor housing conditions in Kembaran, such as unscreened windows, eave gaps, lack of ceilings, create mosquito entry points. Combined with high indoor biting (83.33%), this explains parallel elevation of both antibodies. Prior studies confirm housing construction significantly modulates malaria risk (Kua and Lee, 2021; Nguela et al., 2020). Age analysis revealed no significant variation in either antigen across age groups. Typically, antibody titers increase with age in endemic settings due to cumulative exposure (Rahim et al., 2023). The absence of this pattern likely reflects the 91.6% decline in malaria incidence (2021–2023), indicating age is no longer a reliable cumulative exposure indicator in low-transmission contexts (Office PH, 2024). In near-elimination scenarios, transmission becomes focal and sporadic, younger individuals may experience comparable or greater exposure than older adults if transmission recurs recently (Coalson et al., 2018).

Based on these findings, it can be hypothesized that PfEBA175 primarily reflects recent transmission requiring active control, whereas PfRh5 serves as a cumulative marker capturing past exposure, including submicroscopic or imported infections. PfEBA175, a merozoite surface protein that interacts with glycophorin A, induces antibody responses that decline relatively rapidly in the absence of ongoing transmission, making it a sensitive indicator of recent exposure. In contrast, PfRh5 is essential for erythrocyte invasion and exhibits limited polymorphism, resulting in more durable antibody responses that persist even after transmission decreases. Previous studies have shown that PfRh5-specific IgG antibodies decay more slowly than those targeting other merozoite antigens and may contribute to longer-term protection (Partey et al., 2018; Tran et al., 2014; Kinyanjui et al., 2007). For example, higher PfRh5-specific IgG levels have been linked with a reduced risk of high-density infections in certain cohorts, supporting the idea that PfRh5 responses could reflect a more durable immune memory (Chiu et al., 2014). Consequently, PfEBA175 highlights micro-hotspots of active transmission, whereas PfRh5 identifies areas with residual or historical malaria exposure (Partey et al., 2018; Kinyanjui et al., 2007; Wu et al., 2020).

The integrated interpretation of PfEBA175 and PfRh5 serology alongside identified risk factors has important implications for malaria elimination programs. Serological assays incorporating both antigens provide high-resolution surveillance tools capable of distinguishing areas with active transmission from populations with historical exposure and vaccine design. Operationally, the complementary dynamics of these markers enhance the identification of transmission heterogeneity. PfEBA175 seropositivity may indicate areas with recent or persistent local transmission, whereas PfRh5 responses likely reflect cumulative exposure, including submicroscopic or imported infections. The combined use of these antigens therefore enables more precise targeting of focal interventions, such as indoor residual spraying, targeted case detection, and context-specific behavioral modification strategies (e.g., livestock management in Sedayu or indoor vector control in Kembaran). From a vaccine perspective, the distinct immunogenic behaviors of PfEBA175 and PfRh5 are complementary. PfRh5 is indispensable for erythrocyte invasion and cannot be genetically ablated by the parasite; monoclonal antibodies targeting PfRh5 inhibit invasion effectively *in vitro* (Byrne et al., 2023). Its consistent immunogenicity, even in low-transmission settings, supports its potential to induce broadly protective immunity across diverse endemic contexts. However, evidence indicating that broader antibody repertoires confer enhanced protection suggests that multivalent vaccine formulations incorporating PfRh5 alongside other merozoite antigens, such as PfEBA175, may provide more durable and comprehensive immunity (Osier et al., 2008). In this sense, the dual-antigen serological data presented here not only map the fading contours of malaria transmission but also point toward strategic antigen combinations for next-generation vaccines capable of sustaining protection through the final stages of malaria elimination.

To conclude, these findings highlight the observed serological patterns of PfRh5 and PfEBA175 and their potential implications in a pre-elimination malaria setting. PfRh5 showed relatively consistent immunogenicity across different transmission intensities, suggesting it elicits detectable immune responses even in low-transmission areas and may reflect a more cumulative measure of exposure. In contrast, the heterogeneous responses to PfEBA175 likely mirror recent or localized transmission events, shaped by village-level behavioral, environmental, and vector-related factors. Together, these patterns underscore the importance of a nuanced, village-specific approach to malaria control and elimination and suggest that integrating both antigens could inform multi-antigen vaccine strategies, targeted interventions in areas with residual malaria transmission, and potential for sero-surveillance development. Further work, including longitudinal, entomological, and more detailed behavioral studies, is needed to confirm the specific drivers of these patterns and to validate the proposed use of these antigens as surveillance biomarker.

## Data Availability

The original contributions presented in the study are included in the article/supplementary material, further inquiries can be directed to the corresponding author/s.
